# No Pills, but Letters. Saul Bellow’s *Herzog*: The Recovery of a Depressed Academic

**DOI:** 10.1007/s10912-022-09738-1

**Published:** 2022-06-10

**Authors:** Jeroen Vanheste

**Affiliations:** Department of Philosophy, Open University of the Netherlands, 6503 GJ P.O. Box 6870, Nijmegen, The Netherlands

**Keywords:** Narrative psychiatry, depression, phenomenology, narrative identity

## Abstract

In this article, I discuss the illness and recovery of the depressed Moses Herzog, the protagonist of Saul Bellow’s novel *Herzog* (1964). Using this novel as a case study, I criticize a one-sided (neuro)biological and drug-based approach to depression. Referring to the hermeneutic anthropology of philosophers like Paul Ricoeur and Marya Schechtman, I argue that the treatment of depression could benefit from a broader approach that takes into account existential and social-cultural factors as well as biological factors. I suggest that narrative psychiatry offers a framework wherein various models of mental illness may be combined in ways that move beyond a pro/contra bio-psychiatry binary. By investigating depression using philosophical ideas and a literary text, this article aims to illustrate how the humanities may contribute to our thinking about depression.

In the treatment of depression today, there are many possible approaches. A dominant approach in psychiatry (often referred to as bio-psychiatry) views the depressed person primarily as a body with a disturbed biochemical balance that can be restored through the use of psychopharmaceuticals. In this view, depression is considered a brain disease that must be treated with pills. But many other approaches are possible, including psychoanalytic ones that accentuate the patient’s early life; cognitive behavioral techniques that concentrate on changing the patient’s attitude towards depression; approaches such as interpersonal therapy and family therapy that focus on the personal environment of the patient; therapies that stress the spiritual dimension, such as expressive therapy and mindfulness; approaches that stress the importance of role patterns and cultural context, such as feminist therapy; and several other ones. According to the view of what is called *narrative psychiatry*, there is no single approach that is the best; there are different plausible “stories” to tell (hence the term narrative psychiatry), and therefore there are different possible ways of treatment. Which approach to choose depends on the particular patient, their condition, and their personal preferences. One patient may have total confidence in pills, while another may be highly afraid of side effects or the danger of addiction and may prefer therapeutic conversations or expressive therapy. Furthermore, different approaches might very well be effectively combined, for example, when a patient is treated with medication and cognitive behavioral therapy. The merit of the work by narrative psychiatrists such as Bradley Lewis and SuEllen Hamkins is that they have shown, both in their books and in their psychiatric practice, the potential of a non-binary approach to psychiatry that combines different ways of looking at and treating patients suffering from psychiatric problems (Lewis 2011; Hamkins 2014).

Ways of approaching and treating psychiatric problems are closely related to perceptions of what a human being is. Depending on the view chosen, psychiatric problems may, for example, be seen as “broken” brains, the result of unconscious conflicts, cognitive distortions, family dysfunctions, or as a combination of these. There is no single correct view, and differing perceptions may complement one another. Surely, human beings are in part bodies with particular chemical balances. However, in addition to being a body with certain (biological, genetic, and neurological) properties, humans are also self-interpreting beings that are part of a *Lebenswelt*.^1^ This is a view of humans put forward in the hermeneutic anthropology of philosophers such as Paul Ricoeur, Charles Taylor, and Marya Schechtman. In order to illustrate the implications of this view for the treatment of depression and to exemplify the ideas of narrative psychiatry, I discuss the case of the depressed Moses Herzog, the protagonist of Saul Bellow’s famous novel *Herzog* ([1964] 2003). According to the criteria of the American Psychiatric Association’s (2013) fifth edition of the *Diagnostic and Statistical Handbook of Psychiatric Disorders* (DSM-5), Herzog suffers from severe depression with manic characteristics.^2^

As mentioned above, a range of possible treatments would be open to Herzog today. Most likely, he would be treated with a mixture of antipsychotics and antidepressants, possibly in combination with temporary admission to a psychiatric hospital. Herzog is in such a bad way that such treatments might very well have been helpful; however, when the novel was written, such drugs hardly existed, and apart from a psychoanalytic approach, no good alternative treatments were available. Nevertheless, Herzog manages to control his depression: he gets a new grip on himself through writing letters about his experiences, thus examining and reinterpreting his life. As we will see below, in fact, Herzog becomes his own therapist, practicing a kind of humanistic psychotherapy *avant la lettre*.^3^

The purpose of this article is threefold. Firstly, by investigating depression using philosophical ideas and a literary text, I aim to demonstrate how the humanities may contribute to our understanding of depression. Secondly, I intend to criticize a one-sided bio-psychiatric approach to depression, arguing that Bellow’s novel may help us reflect on a broader and more subject-oriented view of psychiatry, in which depression is approached in the context of biological as well as psychological, existential, and socio-cultural factors. Thirdly, the article aims to discuss the main tenet of narrative psychiatry, namely that no single approach is best in all cases. For a person like Moses Herzog, humanistic therapy might be a good choice, although alternative or additional treatments (in particular, a period of supportive medication) could also be wise and helpful. However, other individuals with different personality traits, values, and personal convictions would probably benefit from different approaches.

## The Significance of the Existential Dimension

The view proposed in this article complies with recent pleas from psychiatrists as well as researchers and philosophers working on theoretical models for psychiatry that more attention be paid to the existential dimension of psychiatry. Psychiatric diseases, they argue, have to do not only with biochemical processes in the brain but also with the way people relate to themselves, their experiences, and their environment. Well-known psychiatrists such as the Dutch Jim van Os have argued for a more patient-oriented approach to depression. On a theoretical level, the possible significance of philosophical approaches like phenomenology and hermeneutics for a better understanding of psychiatric diseases such as depression has been pointed out. Whereas natural science focuses on the human body as an object and thus on explaining physical processes, phenomenology and hermeneutics focus on the patient’s subjective experience of the disease and thus on a better understanding of what it is like to be depressed. It is not a question of one approach being better than the other; because of their different orientations, the two may very well be able to complement one another in a fruitful manner. In this context, the work of philosopher Matthew Ratcliffe is important: in *Feelings of Being* (2008) and *Experiences of Depression* (2014), for example, Ratcliffe examines phenomenologically what it means to be depressed, using philosophical tools and concrete patient experiences. Also interesting is the work of Sanneke de Haan, a philosopher who proposes an “enactivist” approach to psychiatry (de Haan 2020). Enactivism is a model that presupposes relations between body and mind, biology and meaning, and person and environment, thus aiming to do justice to the biological, psychological, socio-cultural, and existential dimensions of psychiatric disorders. Enactivism is an extension of the bio-psychosocial approach, as it takes into account much more explicitly the existential dimension and the ability to *relate* to ourselves, our experiences, and our situation.

## Bellow’s Novel *Herzog*

Saul Bellow was highly interested in timeless questions about the human condition and the interactions between humans and their environment. In 1976, he received the Nobel Prize for Literature for the way in which he exposed his ideas about humans and human culture in his novels: the committee praised both his “human understanding” and adherence to “the realm of values in which man becomes human” as well as his “subtle analysis of contemporary culture” (Atlas 2000, 461). His best-known and most-loved novel is *Herzog*. Despite the book’s complexity (almost the entire novel is set in Herzog’s head, where memory, imagination, reflection, and analysis mingle), it became a public favorite and an unusual sales success; it was translated into many languages and millions of copies were sold. *Herzog* is a fictional account of depression, largely based on Bellow’s personal experiences.

“If I am out of my mind, it’s all right with me,” thus *Herzog*’s opening line (Bellow [1964] 2003, 3).^4^ Moses Herzog is 47 years old and is experiencing a severe crisis. His second marriage has just broken down: his wife Madeleine had a secret affair with his best friend Valentine and kicked him out of the house. Herzog has two children he hardly ever sees; an academic career that had a promising start but has become stuck; financial problems; a country house with an incalculable amount of overdue maintenance; and a host of other difficulties. Enough to make a man despair, Herzog indeed has a very hard time: “His face revealed what a beating he had taken” (6). According to Madeleine, Herzog’s sanity has collapsed (4). She speaks of “a dangerous mental state” (59) and “a sick man, exceptionally neurotic, perhaps even hopeless” (60). Herzog describes himself in similar terms. “His eccentricities had him in their power” (14), he says: “He had moments of sanity, but he couldn’t maintain the balance for very long” (101). He claims to have “symptoms of disorder” (38), calls himself a “lunatic” (117), and repeatedly describes his condition as that of a depressive person (6, 156, 251).

Madeleine sends him to Dr. Edvig, a psychiatrist who talks to Herzog four afternoons a week for some time. Edvig describes Herzog as “reactive-depressive” (60) and warns Madeleine that he is worried about Herzog’s condition (326). Edvig is an old-school psychoanalyst: he talks with his patients while they are lying on a sofa. In the novel, Edvig is portrayed as a charlatan who is more interested in the beautiful Madeleine than in his patient. He is of no use to Herzog, and, as we will see below, the latter will have to save himself. In what now follows, I will discuss what would happen if Herzog should visit a psychiatrist today: What diagnosis would he get, and what treatment would be started? Even though Moses Herzog is a fictional character from a novel written over half a century ago, a thought experiment like this may help to enhance our understanding of the experience and treatment of depression, as will be shown below.

## Moses Herzog’s Condition According to DSM-5

To determine whether a person is depressed, psychiatrists today usually use the DSM-5 criteria. Some of these are psychological; others are behavioral or functional. First, the patient must show at least five of the following nine symptoms on a nearly daily basis: (1) persistent feelings of somberness; (2) loss of interest in and enjoyment of things; (3) a marked change in weight; (4) difficulty in sleeping; (5) psychomotoric agitation, either excessive restlessness or inactivity; (6) fatigue or loss of energy; (7) feelings of guilt or worthlessness; (8) difficulty in concentrating; and (9) thoughts of death and/or suicide. A second criterion is that the symptoms exhibited cause suffering and impaired functioning (social, occupational, or otherwise). Furthermore, these symptoms must not be caused by other factors, such as a somatic disorder or medication use.

How does the situation of Moses Herzog relate to this list? It appears that he meets the DSM-5 criteria for depression. Herzog shows symptoms 1, 4, 7, 8, and 9 to a clear or even high degree and symptoms 2 and 6 to a slightly less pronounced degree. Herzog’s basic mood is one of a deep gloom (symptom 1), verging on despair: “There is someone inside me,” he thinks; “I am in his grip.… He will ruin me” (14). He is afraid that he will “fall apart” (14) and would prefer to “creep into hiding, like an animal” (31). Herzog also suffers from insomnia (symptom 4) and takes sleeping pills “to preserve himself” (16, 117). These are no longer effective, however, and he walks about drugged (88), sometimes more like a zombie than a human being: “His eyes were swollen and his head asleep, but his anxious heart beat faster than ever” (117). As a result of his insomnia, Herzog feels deadly tired (symptom 6): he is “careworn, busted” (298). Another consequence of his insomnia and fatigue is a loss of concentration and disorientation (symptom 8): he is “confused … feverish, damaged … and shaky” (38). In short, he is “a man deeply preoccupied” (9).

Herzog suffers from severe feelings of guilt (symptom 7): towards his first wife Daisy, whom he has treated badly; towards his children, to whom he is barely of any use; towards his academic vocation, which he has let go to waste; towards his family and friends, whom he neglected; towards his girlfriend Ramona, who has been very good to him in his troubling condition, without getting anything in return; and towards himself, because he has been reluctant to confront his problems. A characteristic description in this respect is the following: “To his son and daughter he was a loving but bad father. To his own parents he had been an ungrateful child. To his country, an indifferent citizen. To his brothers and his sister, affectionate but remote. With his friends, an egotist. With love, lazy.… With his own soul, evasive” (7). Given all this, it is hardly surprising that a “perpetual thought of death” (38 [symptom 9]) takes place in Herzog’s mind. At the same time, he feels guilty about having such a thought, because it is “a sin” for him to turn away from life; he remembers the biblical advice “Drive your cart and your plow over the bones of the dead” (38). Herzog’s obsession with death takes the form of confused notes, such as “Death—die—live again—die again—live. No person, no death” (5). It also manifests itself as a fascination for the philosophy of Heidegger—and its *Angst* and *Sein zum Tode*—and especially for that of Heidegger’s successors, the existentialist philosophers like Sartre, for whom, according to Herzog, “the one true God is Death” (315): “Modern philosophers would like to recover the old-fashioned dread of death” (296). Eventually, as we will see below, Herzog will come to reject what he calls the “Wasteland outlook” (82) and overcome his fear; at the height of his depression, however, he suffers from “that infantile terror of death that had bent and buckled his life into these curious shapes” (289).

Herzog thus shows many symptoms of depression. He also satisfies the second criterion mentioned above, as the exhibited symptoms actually cause serious suffering and impair his professional and/or social functioning. His academic career has come to a halt, and he is completely stuck with his book on the interactions between Christianity and Romanticism. His social life is also largely at a standstill. He suffers pain (252) and feels terrible: “his heart is *contemptibly* aching,” and he “would like to give this heart a shaking, or put it out of his breast” (182, italics in original). His is “an injured heart, and raw gasoline poured on the nerves” (219); he lets “the entire world press upon him” (219). “You eat yourself alive,” a friend says to him (236). The situation is of a chronic nature, and suffering has become a “habit” (213). Herzog experiences his condition as hopeless: “I fall upon the thorns of life, I bleed. And then? I fall upon the thorns of life, I bleed. And what next?… I fall upon those same thorns” (225).

## Herzog’s Manic Behavior

But there is even more going on with Herzog. His depressive complaints are accompanied by behavior that exhibits manic characteristics. In order to determine to what extent this is indeed the case, we again consult the DSM-5, where the following criteria are mentioned to determine a manic episode: First, there must be an abnormally and persistently elevated, expansive, or irritable mood and an abnormally and persistently elevated level of activity or energy. In addition, three or more symptoms from the following list should be present: (1) a bloated sense of self-esteem or grandiosity; (2) a reduced need to sleep; (3) a high level of talkativeness or speech impediment; (4) a flight of thought or a feeling of having agitated thoughts; (5) increased distractibility due to irrelevant external stimuli; (6) increased activity (social, work-related, or sexual) or psychomotoric agitation; and (7) engaging in dangerous activities (such as financially or sexually risky behavior). The other criteria for a manic episode include (as with depression) impaired (social, occupational, or other) functioning and the condition that the problems are not caused by other factors such as a somatic disorder or medication use.

Moses Herzog, indeed, clearly meets these criteria for a manic episode. There is abundant evidence of abnormal excitability and activity. In fact, he shows all seven of the symptoms mentioned above and symptoms 1, 3, 4, and 7 to a very high degree. Concerning his self-esteem (symptom 1), Herzog is described several times as a vain and narcissistic person (6, 215). He is aware of this and feels guilty about it; indeed, he calls himself “lazy” in love, a “bad father,” an “ungrateful child,” and “an egotist” (7). When his psychiatrist Dr. Edvig mentions, in a conversation, characteristics such as pride, anger, competitiveness, the inability to endure criticism, hostile projections, and delusions, Herzog thinks to himself: “It’s all there—all!” (86).

Herzog’s thoughts constantly take flight (symptom 4) and “are shooting out all over the place” (16); they “plunged and thundered with endless—infinite!—hungry, electrical power … with inexhaustible energy” (181). He broods over all sorts of things: over personal matters such as his ex-wives and children, his youth and his friends, but also over the historical study of ideas he is writing and over the state of contemporary American and Western culture. Herzog’s days are characterized by a “near-delirium and wide-ranging disordered thought” (303). He is tormented by “the extreme violence of thought and feeling” (237), which sometimes literally takes on a violent character. He regularly fantasizes about revenge: he “wanted to murder Madeleine. Yes, he was capable of killing her” (170), as “now his rage is so great and deep, so murderous, bloody, positively rapturous, that his arms and fingers ache to strangle them [Madeleine and Valentine]” (239). Herzog has the uncontrollable need to express his constantly grinding thoughts. This manifests itself partly as an urge to speak (symptom 3) but much more as an urge to write; as we shall see below, Herzog gives shape to his flight of thought in an endless series of notes and letters.

But Herzog’s mania does not stop at thoughts and also manifests itself in his actions, which often take on dubious and sometimes even dangerous forms (symptom 7). There is talk of Herzog being committed to an institution because he sometimes behaves like “a mad dog” (40) and could become a danger to himself and others. He recognizes this himself: he says that he behaves “quixotic” (311); describes his mood as “eager, grieving, fantastic, dangerous, crazed and, to the point of death, ‘comical’” (102); and acknowledges that he is “in the grip of that eccentric, dangerous force that had been capturing him” (234). At an important point in the novel, Herzog thinks to himself: “Characteristically, he was determined to act without clearly knowing what to do, and even recognizing that he had no power over his impulses” (262). In most cases, his impulsivity is limited to relatively innocent things. For example, he buys clothes he does not need and cannot afford, and he travels to a friend’s summer home in Martha’s Vineyard (because “Seashores are good for madmen” [31]), only to return home immediately upon arrival because he cannot bring himself to be there and talk to his friends. But Herzog’s recklessness sometimes takes on more dangerous forms. While visiting his stepmother in Chicago, he secretly takes a bundle of old ruble bills and his deceased father’s pistol. He then drives to Madeleine and Valentine’s house in a state of great excitement: “The thread of life was stretched tight in him. It quivered crazily.… It did not seem illogical that he should claim the privilege of insanity, violence. It’s not everyone who gets the opportunity to kill with a clear conscience” (276–277). Once at Madeleine and Valentine’s house, Herzog calms down and, after a while, drives away. But the next day, his manic activity starts anew: “Blood had burst into his psyche, and for the time being he was either free or crazy” (288). He picks up his daughter for a day trip but does not pay attention in traffic and gets involved in a car accident. The girl remains unharmed, but he gets slightly injured and faints. He still has the rubles and the gun in his pocket, which causes all kinds of complications when the police arrive. Everything ends well, but the thread on which Herzog balances is thin: “Running to Chicago to protect his daughter, he almost killed her” (310).

In summary, the DSM-5-based diagnosis of Moses Herzog is as follows: Herzog has severe depression with manic characteristics. In many patients with bipolar disorder, manic and depressive episodes can last for extended periods; however, in the case of Herzog, there is a so-called mixed mode, in which the manic and depressive episodes can alternate very quickly, sometimes even within a day. Based on this diagnosis and the list of symptoms mentioned above, a psychiatrist today would probably prescribe a combination of antidepressants, antipsychotics, and sleep medication. A few weeks in a psychiatric hospital would possibly also be advised until the medication begins to work properly and Herzog calms down so that his manic behavior no longer poses a danger to himself or others. As we have seen above, many alternative options (such as expressive therapy, cognitive behavioral therapy, or mindfulness) exist, and a narrative psychiatrist would carefully consider which treatment(s) to choose in consultation with the patient. However, most psychiatrists today would likely go for the medicinal approach, seeing other treatments as only serving a secondary and supportive role—if they were to be considered at all.

## Herzog’s “Treatment”

From a (neuro)biological psychiatric perspective, symptoms like Herzog’s are first and foremost seen as manifestations of a physical (brain) disease. Psychiatric medication is prescribed to make the patient calmer (less confused and/or less anxious) and to restore a disturbed biochemical balance (such as a serotonin deficiency). But at the time Bellow’s novel was published, such medication was hardly available. Herzog has conversations with a psychiatrist who works along psychoanalytic lines, but this man is hardly of any use to Herzog. Despite the lack of serious psychiatric or medicinal help, however, Herzog manages to overcome his misery in the course of the novel. How does he do that?

Herzog refuses to wallow in self-pity and disillusionment and does not flee into booze or other stimulants; instead, he confronts himself. He fights back and does so in his own particular way: he writes letters, an endless series of letters, “to everyone under the sun” (3). He scribbles them down without ever sending them. Initially, many of his letters are rather confused. For example, he writes weird notes, such as “It has been reported that several teams of Russian Cosmonauts have been lost; disintegrated, we must assume. One was heard calling: ‘SOS—World SOS’” (14). Many letters are addressed to people in his own immediate environment and serve as an outlet for his emotions. For instance, he writes to his ex-wives, children, mother-in-law, psychiatrist, himself (“Dear Moses E. Herzog, Since when have you taken such an interest in social questions, in the external world?” [75]), and the dead (“Dear Mama, As to why I haven’t visited your grave in so long” [14]). Other letters have a more intellectual content and are addressed to famous philosophers, including Montaigne, Rousseau, Kant, and Nietzsche (“I also know you think that deep pain is ennobling … and there you have me with you” [347]). A third category of letters has a spiritual character and is addressed to religious thinkers—and even to God (“How my mind has struggled to make coherent sense … I have desired to do your unknowable will, taking it, and you, without symbols” [354]).

Herzog’s writing, at first sight, appears to be the scribbling of a confused, or sometimes even insane, person but is essentially an ongoing self-analysis, a Socratic self-examination: “Late in spring Herzog had been overcome by the need to explain, to have it out, to justify, to put in perspective, to clarify, to make amends” (4). With the help of these letters, he tries to order his thoughts, control his emotions, and reinterpret himself into a comprehensible story. The letters, which gradually become more coherent and clearer, are his private form of therapy. While writing, Herzog searches for direction and tries to reinvent himself: “A loving brute—a subtle, spoiled, loving man. Who can make use of him? He craves use. Where is he needed? Show him the way to make his sacrifice to truth, to order, peace. Oh, that mysterious creature, that Herzog!” (335).

At the beginning of his crisis, Herzog is a misanthropist, furious at all those who have betrayed him and hiding from the world in books and ideas—and constantly writing letters. But, although this helps him to understand himself better, reading books and writing letters, in itself, offers no solution. Above all, it is essential that Herzog shakes off his vanity and complacency and takes account of the social connections that have shaped his life; for, although not unsympathetic, he is an egocentric man, barely involved with his children, family, and friends, and difficult to get along with. He is demanding, suspicious, short-tempered, presumptuous, gloomy, a brooder, vain, and masochistic—not an easy man to live with. He realizes this and feels he must account for it: “He was in pain. He should be. Quite right” (252). Therefore, more important than the books and letters is remembering his youth, living in a poor family on the shabby Napoleon Street in Chicago, where his mother died of cancer when he was 16 years old and his father failed in one job after another. The memories of the bond he had with his parents, brothers, and childhood friends and of life in the city and community at that time are important building blocks in this process of self-investigation. “I am still a slave to Papa’s pain” (163), Herzog, for example, realizes.

Thanks to this process of writing, remembering, and self-reflection, Herzog’s understanding of himself and of his shortcomings increases. Gradually, he starts to think a little milder about Madeleine and Valentine and begins to appreciate their good sides. More generally, he becomes less self-absorbed and more aware of his former self-pity and self-esteem: “Herzog smiled at this earlier avatar of his life, at Herzog the victim, Herzog the would-be lover, Herzog the man on whom the world depended for certain intellectual work, to change history, to influence the development of civilization” (115). Towards the end of the novel, he reconnects with the outside world, including his brother Will, his girlfriend Ramona, and some local residents. Realizing how much he loves his children, he now understands that “a man does not need happiness for *himself* ” (315, italics in original). He intends to become a more active father and also seems to find a renewed bond with nature. At the end of the novel, Herzog lies on the couch in his ruinous country house. He waits for Ramona and realizes his writing impulse is over: “At this time he had no messages for anyone. Nothing. Not a single word” (371). Herzog has conquered the turmoil in his head and is returning to the world.

## Bellow’s Perception of Human Beings

In his novels, Bellow stages humans as self-interpreting beings. Surely, we are partly determined by our biological properties and natural predisposition; at the same time, however, our lives are also shaped by the way we interpret ourselves and the world around us. This has to do with things like values, ideas, and intentions or, in short, with the perspective from which we view the world. In addition to being objects with physical (biological, genetic, and neurological) properties, humans are also subjects that cannot be grasped through the objectifying approach of the natural sciences. This conception of humans can be found everywhere in Bellow’s work. For example, Herzog is convinced that “a man is somehow more than his ‘characteristics’” and that “a Life is something more than such a cloud of particles, mere facticity” (289). At the low point of his crisis, he looks at his face in the mirror and thinks to himself, “My God! Who is this creature? It considers itself human. But what is it?… A desire. Where does it all come from?” (239). At the end of his spiritual journey, lying in a hammock in the garden of his country house, Herzog has found a new balance, but the mystery remains as big as it was:

I look at myself and see chest, thighs, feet—a head. This strange organization, I know it will die. And inside, something, something, happiness … That leaves no choice. Something produces intensity, a holy feeling, as oranges produce orange … this intensity, doesn’t it mean anything? Is it an idiot joy that makes this animal, the most peculiar animal of all, exclaim something? And he thinks this reaction a sign, a proof, of eternity? And he has it in his breast? But I have no arguments to make about it. (370)

Bellow believed in the existence of an invisible dimension in human life and transferred that belief to the characters in his novels. “Something,” a “desire,” a certain “intensity,” a “holy feeling,” an “idiot joy”: it remains somewhat vague and mysterious and cannot be concretized or proven, but Herzog believes it exists. “And what about all the good I have in my heart—doesn’t it mean anything?”, he asks himself, to which he answers, “this good is no phony. I know it isn’t” (225). These intuitions and beliefs of Herzog are similar to those of his creator. In an interview, Bellow worried about the attempts to rid human life of its mystery: “Science with the aid of modern philosophy—what we call the positive outlook—has driven the ‘invisible’ into the dark night where enlightenment says it belongs” (Cronin and Siegel 1994, 222). Like Bellow, Herzog is critical of the pretensions of that “positive outlook” and of scientific models that claim to solve the human mystery: “human life is far subtler than any of its models” (Bellow [1964] 2003, 295). Related to this, Herzog rejects any rigid materialism and determinism; although human beings are obviously shaped by their biological properties and socio-economic environment, these can never fully determine humans. Humans cannot be calculated and remain “the composite, the mystical achievement of natural forces and his own spirit” (168). When Herzog looks at the stars in the garden of his country house, he realizes it is humans who form matter and not the other way around: “When he opened his eyes in the night, the stars were near like spiritual bodies. Fires, of course; gases—minerals, heat, atoms, but eloquent at five in the morning to a man lying in a hammock, wrapped in his overcoat” (3).

Rather than powerless victims, Bellow portrays his characters as humans who are, to a certain extent, free to shape themselves and capable of taking responsibility for their own lives. No matter how hard life may be, self-pity or resignation is never an option. “I’m not going to be a victim. I hate the victim bit” (90), Herzog says to a friend. He suffers, but he has the inner reserves to get through it, and he is convinced that it is his duty to do so: “He must live. Complete his assignment, whatever that was” (251). In an interview with Sanford Pinsker, Bellow said that Herzog’s task, which is to better understand himself and the world around him, is, in fact, that of us all. “I think in a world where people had better have an ‘earnest’ grip on reality in order to survive, it becomes a mental-moral project to understand your surroundings, yourself, and the society you live in” (Cronin and Siegel 1994, 97). “*I* am Herzog,” he realizes; “I have to *be* that man. There is no one else to do it” (Bellow [1964] 2003, 74, italics in original).

Bellow was certainly not naively optimistic when it came to human freedom and agency. His view of humankind was strongly influenced by modern science and post-war philosophy and has therefore left behind any naivety with regard to human autonomy; the characters in his novels are strongly marked by their natural disposition and (social, economic, and cultural) environment. Nevertheless, Bellow maintained a humanist orientation. Herzog, for example, remains present as an individual—as a self that directs his existence—although his childhood experiences and family background have a major impact on him, and he does have ambiguities and contradictions. In fact, he realizes this very well, contemplating that “modern character is inconstant, divided, vacillating,” for it lacks the “stonelike certitude” of earlier ages (125). Although Herzog’s autonomy is thus problematized, a certain core of his personality remains; this core, as we will now see, can be understood narratively.

## Life As a Story: the Narrative Self

Bellow’s portrayal of Moses Herzog illustrates the hermeneutic anthropology advocated by philosophers such as Paul Ricoeur, Charles Taylor, and Marya Schechtman. In their view, humans are, at least partly, constituted by their (self-)interpretations. These interpretations have to do with our natural characteristics and embodiment, as we are biological beings, but also depend on our environment: the biographical, social, historical, and cultural factors that form the context of our lives. Self-interpretation and self-understanding do not take place in a void. “A human being alone is an impossibility,” says Taylor, “self-interpretations which define [an individual] are drawn from the interchange with the community” (1985, 8). Ricoeur also stresses that “To say self is not to say I” (1992, 18).

Essential to this perception of humans is that it is not so much concerned with *what* a human is (for example, a set of biological, genetic, and neurological characteristics) as it is with *who* a human is—namely, someone with a certain past, living in a certain social and cultural environment, and with certain values, motivations, goals, and intentions. A self is not an object *in* the world but a perspective *on* the world. Since a subject and its self-interpretation are not a visible part of the empirical reality, the notion of a self is meaningless from a scientific point of view. As Taylor writes, “What it is to possess a liver or a heart is something I can define quite independently of the space of questions in which I exist for myself, but not what it is to have a self or to be a person” (1985, 4).

Important in this context is Ricoeur’s distinction between identity as *idem* and identity as *ipse*, where *idem* refers to identity as immutability (for example, biological properties such as our genetic code) and *ipse* refers to identity as subjectivity. *Idem* refers to *what* we are; *ipse* refers to *who* we are. Considered as *ipse*, we do not remain identical in the sense of a physical or psychological immutability but in the sense of a developing personality. *Idem* belongs to “the category of events and facts” (Ricoeur 1991a, 193), whereas *ipse* belongs to “the mode of being of [Heidegger’s] *Dasein*” (Ricoeur 1991a, 191), a mode of being in which no assumption is made “concerning some unchanging core of the personality” (Ricoeur 1992, 2).

The philosophers mentioned propose to approach human identity as a kind of story. After all, our (self-) interpretations resemble stories, which by their nature match the form of human life; they take place in time and are about the actions of characters with certain backgrounds and intentions. Ricoeur says that stories can express “the very structure of human acting and suffering” and points to “the inestimable value of narrative for putting our temporal experience into order” (1991b, 28, 31).

Every life knows its tensions (such as conflicting loyalties) and contradictions (such as a personality that changes over time). Human identity is dynamic and characterized by changes and developments. “The issue of our condition can never be exhausted for us by what we are,” writes Taylor, “because we are always also changing and becoming” (1989, 46–47). What we need in our self-interpretation, therefore, is “a notion of how we have become, and of where we are going” (Taylor 1989, 47). In this respect as well, stories can be helpful. In a story, I can connect my past experiences with my present situation (my social and cultural context and my values and beliefs) and my goals for the future. With the help of a story, I can try to understand my life as a whole, thus trying to get a grip on my identity and existence. “It is through story that we find or devise ways of living bearably in time,” writes Taylor (2016, 319).

Through my story, I can tell myself and others “who I am.” By approaching our life as a personal story, we can try to transcend the ambiguity and vicissitudes of our existence and see ourselves and our life as a unity; a unity that is not biological but thematic, moral, and existential. It is by putting together our own story that we truly *become human* and transcend what we, of course, are as well: a biological being. Ricoeur, Taylor, and Schechtman speak of a *narrative identity*; that is, the self, as it comes about through interpretations and is expressed in a story. When we talk about a narrative identity, we refer to the self as *ipse*.

The idea of narrative identity as a hermeneutic view of the self implies a belief in a certain degree of human freedom and, therefore, responsibility. “Narrativity serves as a propaedeutic to ethics,” says Ricoeur (1992, 115) in this regard. However, this freedom is always situated and, therefore, relative because it inevitably depends on all kinds of circumstances you hardly can control, such as your physical health and the social and cultural context in which you are brought up. Ricoeur says that “we can become our own narrator … without being able to become the author” (1991b, 32). In this context, Marya Schechtman distinguishes three roles we play: character, critic, and author. As a character, our story is determined by our circumstances and environment; as a critic, we interpret what happens to us and reflect on it; and as an author, we may try, to a certain extent, to rewrite our own story (Schechtman 2011, 412–414).

In summary, philosophers such as Taylor, Ricoeur, and Schechtman regard humans as beings who ceaselessly interpret themselves and their world. Our interpretations depend on our inner *cogito* and imagination as well as on our embodiment and *Lebenswelt*. In articulating our interpretations and understanding of human identity, stories can play a crucial role in many ways: by justifying choices we made; making tensions and contradictions understandable; indicating which values are important to us; showing who we (think we) are and who we want to become—or, in short, by seeing our life in its coherence and meaning. “We must inescapably understand our lives in narrative form,” writes Taylor (1989, 52), and Ricoeur agrees that “A life is no more than a biological phenomenon as long as it has not been interpreted [in a story]” (1991b, 27–28). Ricoeur describes human life as “*activity and passion in search of a narrative*” (1991b, 29, italics in original). According to him, our existential state is “entangled in stories,” and interpreting and narrating our lives is a way to respond to Socrates’ call to investigate our lives (Ricoeur 1991b, 30–31).

## A Humanistic Approach Toward Depression

Bellow’s conception of human beings fits seamlessly with the hermeneutic anthropology that interprets the human self in a narrative way. In his Nobel Prize acceptance speech, Bellow fiercely opposed the so-called “death of the individual,” which is proclaimed by philosophical and scientific views that state there is no such thing as a self since we are little more than pre-programmed matter or a product of our environment and circumstances. “Can it be that human beings are at an end?” he asked, answering the question in the negative: science and theory can only give a limited and objectified picture of us while we long for “a broader, more flexible, fuller, more coherent, more comprehensive account of what we human beings are, and what this life is for” (Bellow 2015, 299–300). The defense of human personality, human agency, and human potential and growth—in an age wherein these humanist axioms are questioned—is central to Bellow’s work. He spoke of himself as an “interpreter of the human heart” (Bellow 2015, 1). He was convinced that we have a self, a personality that defines our humanity and forms the key to the meaning of our life. Yes, science and philosophy have convincingly shown that this concept of a self is problematic. But their conclusions have been far too drastic. There is no need, thus, for Bellow to declare the death of the individual, for this is a form of misanthropy that removes all depth from life (2015, 238). Natural science cannot answer the major questions about man, identity, and meaning: “He is something. What is he? The mystery increases, it does not grow less” (Bellow 2015, 195).

Bellow’s novel about Moses Herzog is a sublime illustration of both Bellow’s criticism of the alleged death of the individual and the hermeneutic anthropology in which a human is seen as a self-interpreting being with a narrative identity. Herzog reinterprets his entire past life, which leads to a better self-understanding and helps him to recover from his depression. Herzog’s letter writing is an example of what the Austrian neurologist and psychiatrist Viktor Frankl called *logotherapy*, a form of humanistic psychotherapy.^5^ According to Frankl ([1946] 2006), the human condition is characterized by a *will to meaning*, and people benefit from giving meaning to their lives by seeing themselves as participants in a story with a certain direction or intention. Herzog’s letters, indeed, play this role for him; “I go after reality with language,” he says to his friend Lucas Asphalter (Bellow [1964] 2003, 296), and he imagines “he was an industry that manufactured personal history” (5).

Through its humanistic orientation, Bellow’s novel warns us against a form of psychiatry that merely treats depression as a brain disease. Undoubtedly, there may be a biological basis for depression, and physical factors—such as hereditary predisposition—and the workings of neurotransmitters and hormones play their part. Fortunately, there are drugs that can improve our biochemical balance and offer relief. However, the depressive patient is not only an object consisting of biological properties but also a self-interpreting subject rooted in a *Lebenswelt*. Bellow’s novel suggests that this dimension is also crucial to the recovery from depression. Yet, the novel certainly does not provide a blueprint for how to deal with depression, for Herzog, in fact, receives no professional treatment at all (Doctor Edvig can hardly be taken seriously) and is largely left to his own devices (namely, his logotherapeutical self-treatment). At times, he walks along the edge of the abyss—for example, when he arrives at the house of Madeleine and Valentine in a manic state armed with a gun or when he has a traffic accident with his little daughter in the car. Whereas modern psychiatry often places all emphasis on pills, there is no medication whatsoever in the case of Herzog, so his “treatment” is too one-sided as well.

## Narrative Psychiatry as a Deeply Human-centered Approach

A reasonable way to deal with depression seems to be to strive for a synthesis of biomedical and personal treatments. In recent years, there have indeed been calls from various quarters for a broader and more person-centered holistic approach to psychiatry, an approach in which, in addition to the use of psychopharmaceuticals, consideration is also given to the patient’s personal background and *Lebenswelt*. Psychiatrists such as Jim van Os question the massive scale on which medication is prescribed, the effectiveness of medication and the lack of information about possible side effects, and, more generally, the one-sided bio-psychiatric approach that is the current paradigm. As mentioned above, philosophers such as Matthew Ratcliffe and Sanneke de Haan have proposed models for psychiatry that make room for the existential dimension of human life, a dimension that de Haan finds largely missing in the bio-psychosocial model (de Haan 2020). In such approaches, serious attention is given to subjectivity, interactions with the *Lebenswelt*, patient experiences, and literary and artistic representations of the experience of depression.

In fact, the idea of combining different possible approaches is also what a narrative psychiatry framework offers. There are several models of mental illness, each one having its own point of view and using its own specific metaphors. Biological models speak of “broken brains” and chemical imbalances; psychoanalytic models speak of unconscious conflicts; cognitive models speak of cognitive distortions or “broken thoughts”; family-oriented models speak of family dysfunctions; and so on. Even holistic models like the bio-psychosocial and enactive ones use a particular point of view, excluding or ignoring others. Central to narrative psychiatry is the belief that there is no single correct or best way to interpret and treat mental health problems. All models for psychiatry offer potential tools for interpreting mental disorders. All of them—the medical as well as the other ones, including holistic models—correspond to particular “stories” and ways of “seeing as” (Lewis 2011, 148); all propose a particular narrative to help people reauthor their stories. The choice of which way to proceed highly depends on the patient’s particular situation and preferences. A narrative psychiatrist helps the patient to identify possible directions (Lewis 2011, 23) and, in a deeply patient-centered approach, considers various possibilities without insisting on a particular right way to proceed. Narrative psychiatry, thus, intends to move beyond a pro/contra bio-psychiatry dichotomy (Lewis 2011, 16). As Bradley Lewis stresses, this absolutely does not imply an “anything goes” relativism, the point being that the prism through which people see themselves and the stories they tell about themselves not only *describe* but also *shape* their lives (Lewis 2011, 44). Referring to Paul Ricoeur, Lewis (2011, 44) mentions that where universal truth may exist in the domain of natural science, we might speak of a “metaphorical truth” in a human context of mental illness: it may be best to choose an approach that does justice to the metaphors through which we see ourselves. If I am someone who, like Moses Herzog, strongly believes in human agency and values the existential dimension of life, a humanistic therapy could be the way to go; whereas, when I firmly believe that we are physical creatures ruled by genes, hormones, neurons, and so on, taking medication probably might be a good idea. In many cases, a combination of therapies (for example, a combination of talking and pills) might be a viable way to go. Logotherapy seemed a very fitting approach for Herzog; however, considering his serious condition, it probably would have been better to take medication for a while, too, in order to avoid the total chaos he now inflicted. Furthermore, for others facing depression, logotherapy might not be a suitable idea at all.

## Conclusion

In accordance with the interdisciplinary approach of the medical humanities, philosophy and literature may help us to reflect on and explore the experience of psychiatric disorders. Using Bellow’s novel and the ideas of philosophers like Ricoeur, Ratcliffe, and de Haan, I discussed the story of Moses Herzog as a case study of depression. Although Herzog only exists in the novel, and although there was no DSM-5 at the time Bellow wrote it, using fictional characters can be highly useful in thought experiments that aim to enhance our understanding of the experience and treatment of psychiatric illnesses. In his book on narrative psychiatry, Bradley Lewis, in a similar way, uses stories by Anton Chekhov and Chitra Divakaruni to express his ideas and explore hypothetical scenarios.^6^ Bellow himself said in this regard that the novel as a genre is pre-eminently capable of showing ideas about humans in “flesh and blood” by shaping these into characters (Bellow 2015, 130).

I have argued that Bellow’s novel, as an account of the experiences of a person with severe depression, can help us to reflect on the shortcomings of current biomedical psychiatry and put us on the trail of more human-centered psychiatry. *Herzog* is an expression of faith in human beings as persons, as self-interpreting subjects rooted in a *Lebenswelt*, a view also expressed in the hermeneutic anthropology of Ricoeur, Taylor, and Schechtman and the enactivist model of psychiatry put forward by de Haan. Herzog reminds us of what we are in danger of forgetting when we agree too easily with a purely biological conception of humans—he shows us what it means to be “A human being! A *mensch*!” (90, italics in original). The humanism of Bellow and of philosophers such as the ones mentioned remains highly urgent in a time when neuroscientific research into *what* we are is considerably more influential than narrative reflections on *who* we are.

I sympathize with an existential humanist approach to depression that includes personal, socio-cultural, and biological factors, and the work of philosophers like Matthew Ratcliffe and Sanneke de Haan is of crucial importance in this respect. At the same time, it has to be acknowledged that human-centered approaches and enactivist models are still no more than particular narratives, albeit holistic ones that avoid reductionist focus on one factor. One problem with human-centered approaches might be that they work best with “strong” characters possessing a certain sense of autonomy and agency. But not everyone is like Moses Herzog, and for other types of patients, alternative views, narratives, and, therefore, ways of treatment will be better options. Herzog benefits from logotherapeutical letter writing, but for others, a biomedical approach or one of the many other available therapies like creative therapy, feminist therapy, or spiritual therapy might very well be a better choice. Narrative psychiatry reminds us that there are many plausible stories to tell and suggests that the decision of which story will be told should not be taken in a doctor-knows-best fashion; rather, it should be taken in a way that respects the values and the lived experience of the patients and honors the way in which they view themselves and their world.

## Footnotes

1 The concept of *Lebenswelt* was used by philosophers such as Husserl and Heidegger to refer to the world of human experience, as opposed to the empirical world that can be scientifically researched. I use the term here to refer to the biographical, social, historical, and cultural factors that form the context of human life.

2 The DSM-5 is used as the standard for psychiatric diagnostics in many countries over the world.

3 Bradley Lewis defines humanistic psychotherapy as a phenomenological approach that views the client as the expert of their own experience, considers human agency to be the final arbitrator of choice and self-determination, and is a person-centered approach that includes a deep respect for the uniqueness of each individual (Lewis 2011, 114).

4 Subsequent references to the novel are included in the text between parentheses.

5 This is the topic of Frankl’s book *Man’s Search for Meaning* ([1946] 2006).

6 See, in particular, Chap. 1 (“Listening to Chekhov”) and Chap. 5 (“Mrs. Dutta and the Literary Case”) of *Narrative Psychiatry* (Lewis 2011).
